# Electrophysiological dynamics of antagonistic brain networks reflect attentional fluctuations

**DOI:** 10.1038/s41467-019-14166-2

**Published:** 2020-01-16

**Authors:** Aaron Kucyi, Amy Daitch, Omri Raccah, Baotian Zhao, Chao Zhang, Michael Esterman, Michael Zeineh, Casey H. Halpern, Kai Zhang, Jianguo Zhang, Josef Parvizi

**Affiliations:** 1 Department of Neurology & Neurological Sciences, Stanford University, Stanford, CA 94304 USA; 2 Department of Neurosurgery, Beijing Tiantan Hospital, Beijing, 100070 China; 3 Department of Neurosurgery, Beijing Neurosurgical Institute, Capital Medical University, Beijing, 100070 China; 4 Boston Attention and Learning Laboratory & Neuroimaging Research for Veterans Center, Veterans Administration, Boston Healthcare System, Boston, MA 02130 USA; 5 Department of Psychiatry, Boston University School of Medicine, Boston, MA 02130 USA; 6 Department of Radiology, Stanford University, Stanford, CA 94304 USA; 7 Department of Neurosurgery, Stanford University, Stanford, CA 94304 USA

**Keywords:** Neuroscience, Cognitive neuroscience

## Abstract

Neuroimaging evidence suggests that the default mode network (DMN) exhibits antagonistic activity with dorsal attention (DAN) and salience (SN) networks. Here we use human intracranial electroencephalography to investigate the behavioral relevance of fine-grained dynamics within and between these networks. The three networks show dissociable profiles of task-evoked electrophysiological activity, best captured in the high-frequency broadband (HFB; 70–170 Hz) range. On the order of hundreds of milliseconds, HFB responses peak fastest in the DAN, at intermediate speed in the SN, and slowest in the DMN. Lapses of attention (behavioral errors) are marked by distinguishable patterns of both pre- and post-stimulus HFB activity within each network. Moreover, the magnitude of temporally lagged, negative HFB coupling between the DAN and DMN (but not SN and DMN) is associated with greater sustained attention performance and is reduced during wakeful rest. These findings underscore the behavioral relevance of temporally delayed coordination between antagonistic brain networks.

## Abstract (web summary)

Brain imaging studies suggest that specific, large-scale, cortical networks show antagonistic activity with one another. Here, the authors studied the dynamics of these networks using implanted electrodes in the human brain, revealing that the coordination of inter-network dynamics on fast time scales relates to fluctuations in attention.

## Introduction

The brain structures important for attention have long been described as a set of discrete components of networks, each of which serves a unique role in cognitive interactions with the sensory environment^1^. The dorsal attention network (DAN) is implicated in goal-directed (top–down) attention^2^, and the salience network (SN) is implicated in stimulus-driven (bottom–up) attention and cognitive control^3,4^. The DAN and SN are activated during a wide variety of task conditions involving externally oriented attention^2,5^. Given functional neuroimaging evidence, these described networks exhibit a distinct activity profile with yet another set of regions that constitute the default mode network (DMN)^6,7^, which unlike DAN and SN structures, tends to show deactivation during conditions involving externally oriented attention^8^.

In addition to task-dependent activity, functional magnetic resonance imaging (fMRI) studies during wakeful rest have shown spontaneous, negatively correlated (or “anticorrelated”) DMN-DAN/SN activity in infraslow (<0.1 Hz) fluctuations of blood-oxygen-level-dependent (BOLD) signals^7,9^—a finding that has remained contentious^10^. Persistence of such continuous (i.e., non-task-evoked) anticorrelated activity would potentially suggest that functionally competing systems, characterized by ongoing switches between internally- and externally biased modes of attention, are an intrinsic property of the brain^11,12^. However, continuous anticorrelated activity between DMN and DAN or SN may be partly dependent on cognitive state, given that the degree of inter-network BOLD anticorrelation dynamically varies over time^13^ and can be associated with variations in task conditions^14^ and fluctuations in behavioral performance^15,16^. Moreover, regional BOLD activity within these networks varies with lapses of attention and self-reported mind-wandering^17–20^.

Importantly, evidence for both task-evoked and task-free antagonistic activity among the DMN, DAN, and SN has relied almost exclusively on fMRI, which offers limited temporal resolution and necessitates data preprocessing that may bias estimates of negative correlations^21,22^. Intracranial electroencephalography (iEEG) in human subjects offers anatomical precision, high temporal resolution, and sensitivity to activity in the high-frequency broadband (HFB, also known as high gamma) range ( 70–170 Hz)—a well-established correlate of the BOLD signal and neuronal population spiking^23–26^. A handful of iEEG studies involving recordings from putative DMN and DAN nodes have shown task-evoked HFB responses that resemble the opposing inter-network (de)activations observed in fMRI^27–30^. In addition, slow fluctuations of HFB power amplitude during wakeful rest were shown to exhibit inter-regional correlated activity (functional connectivity) within networks, including DMN and DAN, with spatial topographic patterns similar to those found with BOLD imaging^31–35^. In particular, strong, and highly focal within-network iEEG correlations have been consistently found for slow (<1 Hz) and infraslow (<0.1 Hz) HFB fluctuations^31–34^.

The extant iEEG evidence provides promising initial electrophysiological validation of antagonistic network interactions during task performance as well as the persistence of within-network correlated activity during wakeful rest. However, critical open questions remain about the behavioral relevance of electrophysiological dynamics of antagonistic networks: do nodes of the DMN, DAN, and SN exhibit distinguishable, systematic profiles of task-evoked responses on the order of hundreds of milliseconds and in specific components of electrophysiological signals? Does dynamic coordination of activity within and between these networks relate to fluctuations in attentional task performance? Does continuous anticorrelated activity of HFB activity between task-responsive DMN, DAN, and SN neuronal populations vary between externally oriented task performance and wakeful rest? To address these questions, here we report a comprehensive iEEG investigation of activity within and interactions among the DMN, DAN, and SN in a large cohort of subjects with electrodes implanted directly within key cortical nodes of these networks. We show that the three networks show distinct profiles and timing of task-evoked electrophysiological activity and that antagonistic inter-network dynamics relate to attentional performance fluctuations.

## Results

### Unique iEEG cohort

We obtained iEEG recordings from a total of 3704 unique recording sites in 31 human subjects (S1–31), included in our main analyses, who were undergoing treatment for focal epilepsy (29 with depth and 2 with subdural electrode recordings) at Stanford Medical Center (*n* = 10) or Beijing Tian Tan Hospital (*n* = 21). Data reported here are from regions void of pathological activity. Given that different patients had distinct epileptic foci (Supplementary Table 1), our group-level analyses ensured that results could not likely be attributed to pathological activity.

**Fig. 1:**
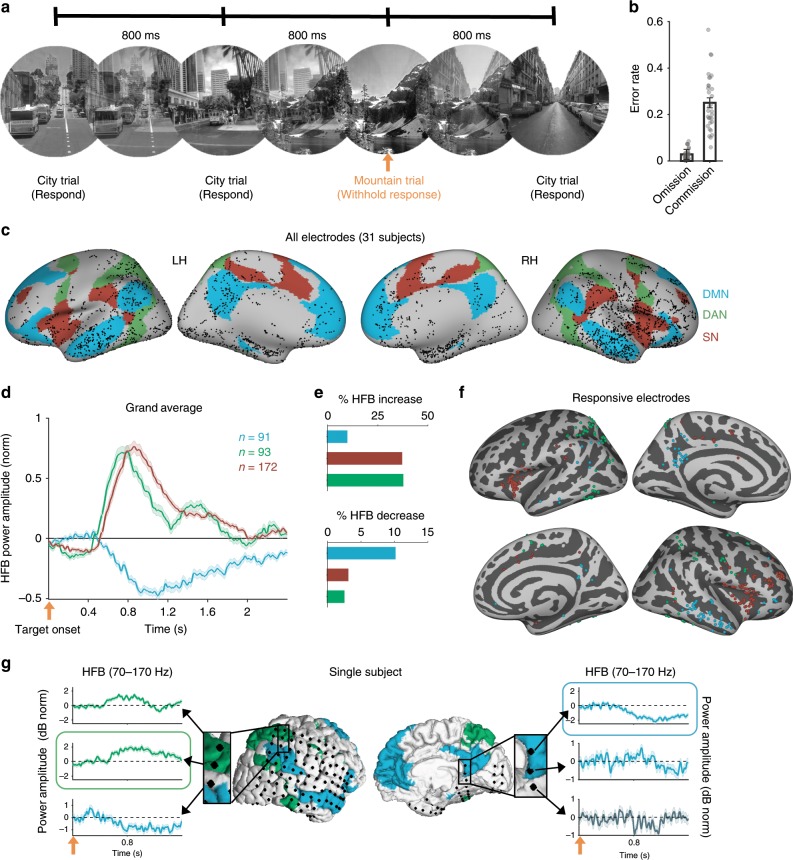
Task paradigm and functional localization of electrode sites in three networks of interest. **a** The Gradual-Onset Continuous Performance Task. City and mountain scene images (from the SUN database^99^) faded continuously from one image to the next every 800 msec. Trial onset (orange arrow) was the time at which stimulus fade-in was initiated. **b** Omission and commission error rates, averaged across runs, in 31 subjects. **c** Anatomical locations of cortical electrode contacts in 31 subjects, projected to fsaverage standard space and overlaying the Yeo atlas’ DMN (blue), DAN (green), and SN (red). **d** High-frequency broadband (HFB; 70–170 Hz) responses during the presentation of mountain (target) stimuli. Time courses show the grand average of the trial-wise means within all responsive electrodes (*p* < 0.05, cluster-based permutation test, corrected for multiple comparisons within networks within subjects) across the entire patient cohort. **e** Relative proportions of electrodes within each network showing significant target-evoked HFB increase (top) and decrease (bottom). **f** Locations of responsive electrodes within each network (fsaverage space). **g** Subdural electrodes plotted on the cortical surface with an overlay of the Yeo atlas’ DMN (blue) and DAN (green) in an example subject. Time series plots illustrate diverging HFB response profiles among neighboring electrodes (peak-responsive electrodes in the dPPC and PMC, respectively, are outlined in green and blue). Where relevant, error bars indicate standard error of the mean.

Subjects performed between four to eight runs (total duration range: 24–48 min per subject, see Supplementary Table 2) of the Gradual-onset Continuous Performance Task (GradCPT), a test of sustained attention that has reliably been associated with opposing patterns of task-evoked DMN versus DAN/SN activity in fMRI studies^18,19,36^. Gradually changing images of scenes were presented every 800 ms, and subjects were instructed to respond with a button press to city (frequent) but not to mountain (infrequent) scenes (Fig. 1a). After excluding subjects with poor behavioral performance (see Methods), analysis of our cohort of 31 subjects revealed rates of omission and commission errors (M ± SD: 2.9 ± 2.3% and 25.1 ± 12.0%, respectively) that were comparable to those previously reported in healthy and clinical populations (Fig. 1b)^18,36^. We performed subsequent analyses within the entire cohort as well as within subcohorts where simultaneous coverage of electrodes was available across the antagonistic networks of interest (see Supplementary Fig. 1 for analysis workflow).

### Functional localization of electrophysiological networks

Each subject had electrode coverage within cortical regions of the DMN, DAN, and SN, as defined based on an fMRI-based population-level atlas of intrinsic networks (Fig. 1c)^37^. To functionally localize electrode sites within the networks, we first analyzed task-evoked electrophysiological activity during trials of infrequent targets (mountains) relative to frequent non-targets (cities) in the GradCPT. Using cluster-based permutation testing^38^, we screened all electrodes for the expected task-evoked decreased HFB power in the DMN and task-evoked increased HFB power in the DAN and SN during the time window of 0–1500 ms post-target onset.

In 29 out of 31 subjects, we successfully identified electrodes with significant HFB responses within at least one of the three networks (Monte Carlo *p* *<* 0.05, cluster-based permutation test, corrected for the number of electrodes within each network within subjects). The grand averages of significant electrodes indicated that transient, increased HFB in the DAN and the SN as well as decreased HFB in the DMN began several hundred milliseconds after target onset and returned to baseline after ~ 1–2 s (Fig. 1d; see Supplementary Fig. 2a for Stanford and Beijing cohorts separately and Supplementary Fig. 3 for individual subject plots). Across all electrodes, the relative proportions within each network showing significant increased target-evoked HFB power (i.e., activation) were 37.3% (DAN), 36.8% (SN), and 9.5% (DMN) (Fig. 1e, top; see also Supplementary Fig. 2b). In contrast, the relative proportions showing significant decreased target-evoked HFB power (i.e., deactivation) were 2.4% (DAN), 3.0% (SN), and 10.0% (DMN) ((Fig. 1e, bottom; see also Supplementary Table 2 for relative contributions of individual subjects). Non-unanimous responses are often seen among electrodes in a region of the brain across subjects but also within the same individual subject, as previously reported^39^. Overall, however, our findings confirm that electrodes with the expected response characteristics based on network identity were more frequently identified than those with the opposite characteristics.

The responsive electrodes were distributed throughout multiple cortical locations within each network (Fig. 1f; see Supplementary Fig. 2c for Stanford and Beijing cohorts separately). Responsive sites were identified within well-described core regions of the networks: these included the dorsal posterior parietal cortex (dPPC), frontal eye fields and area MT+ within the DAN, the dorsal anterior insular cortex (dAIC), mid-cingulate cortex, and dorsolateral prefrontal cortex within the SN, and the posteromedial cortex (PMC), medial prefrontal cortex and angular gyrus within the DMN. The correspondence between HFB response profile and intrinsic network identity could be illustrated in cases where subdural electrodes densely covered areas near network boundaries (Fig. 1g). Taken together, the correspondence between standard intrinsic network boundaries and iEEG response profiles suggested that the general functions of the DAN, SN, and DMN were preserved within subjects.

### Frequency band specificity of electrophysiological responses

**Fig. 2:**
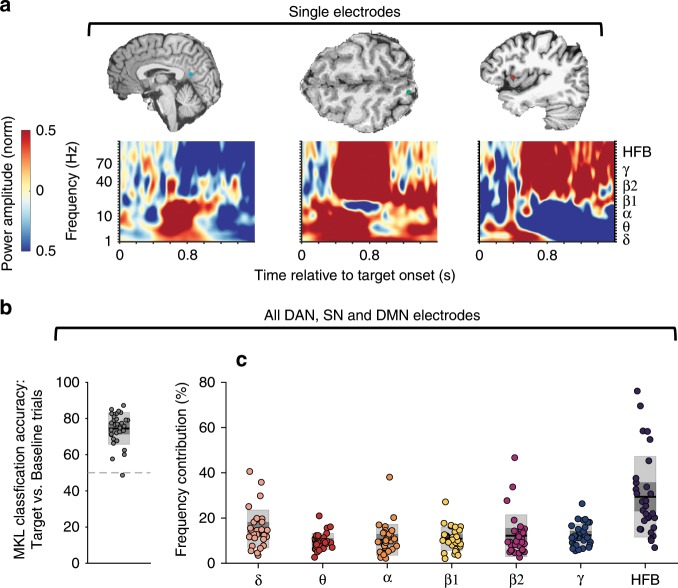
Frequency-specific contributions to task-evoked responses. **a** Example time-frequency plots highlighting spectral changes during correct omissions (mean across trials) for depth electrodes located within key nodes of the DAN (dorsal posterior parietal cortex), SN (dorsal anterior insular cortex), and DMN (posteromedial cortex). **b** Classification accuracy (correct omissions versus correct commissions) in 31 subjects based on multiple kernel learning analysis (full model with seven frequency ranges and all electrodes within the DAN, SN, and DMN). **c** Contributions of the power amplitudes of distinct frequency bands to the classification accuracy shown in **b**. In box plots, center lines indicate mean, dark gray areas indicate 95% confidence interval, and light gray areas indicate SD.

Although we have focused so far on neural activity within the HFB range, spectrograms of individual electrodes indicated that task-evoked responses included lower frequency components (Fig. 2a). Thus, to assess the degree to which task-evoked responses were specific to the HFB range, we adopted a multivariate approach (multiple kernel learning; MKL) to decompose the relative contributions of the power amplitudes of distinct frequency bands^40^. Specifically, for target (mountain) and baseline (city) trials with correct behavioral responses, in the time window of 0–1500 ms post-trial onset, we defined kernels, or pair-wise similarity among all trials (target and baseline) of frequency-specific power amplitudes. These kernels were defined for each electrode that was within at least one of the networks (DAN, SN, DMN) and for the power amplitudes of seven frequency bands [*δ* (1–3 Hz), *θ* (4–7 Hz)*, α* (8–12 Hz)*, β1* (13–29 Hz)*, β2* (30–39 Hz)*, γ* (40–70 Hz), HFB (70–170 Hz)]. Feature selection was performed on the kernels (i.e., of size seven frequencies x number of electrodes within each subject) such that variability in the relative contribution of each frequency band could be quantified (see Multiple Kernel Learning Analyses of Distinct Frequency Bands in Methods). As we considered all electrodes that were anatomically within the DAN, SN, and DMN within the MKL models, this data-driven analysis was independent of and complementary to our aforementioned screening procedure for responsiveness within the HFB range.

Using 10-fold cross-validation to classify trials as correct omission versus correct commission, we found classification accuracies with levels above chance (balanced accuracy: M±SD = 74.5 ± 8.7%; correct omission accuracy: 73.8 ± 9.5%; correct commission accuracy: 75.2% ± 8.3) (Fig. 2b). A repeated-measures ANOVA revealed a significant effect of frequency band on contribution to classification accuracy (*F*_*6,180*_ = 15.2, *p* = 5.1 × 10^−14^). Across frequencies, HFB features had the highest mean contributions to classification accuracy, and these HFB contributions were significantly higher than those of all other frequency bands (*p*_*FDR*_, paired *t* tests: HFB vs. δ: 0.002; HFB vs. θ: 0.0001; HFB vs. α: 0.0001; HFB vs β1: 0.0001; HFB vs β2: 0.0004; HFB vs. *γ*: 0.0001) (Fig. 2c). Thus, across subjects, the HFB signal most consistently provided information about functional activity underlying cognitive processing within the DAN, SN, and DMN. Given these findings, as well as extensive prior evidence showing that HFB activity is a well-established correlate of the BOLD signal and neuronal population spiking^23,24,41^, we focus our central further iEEG analyses on HFB signals but also consider other frequency ranges.

### Distinct HFB response timing among networks

**Fig. 3:**
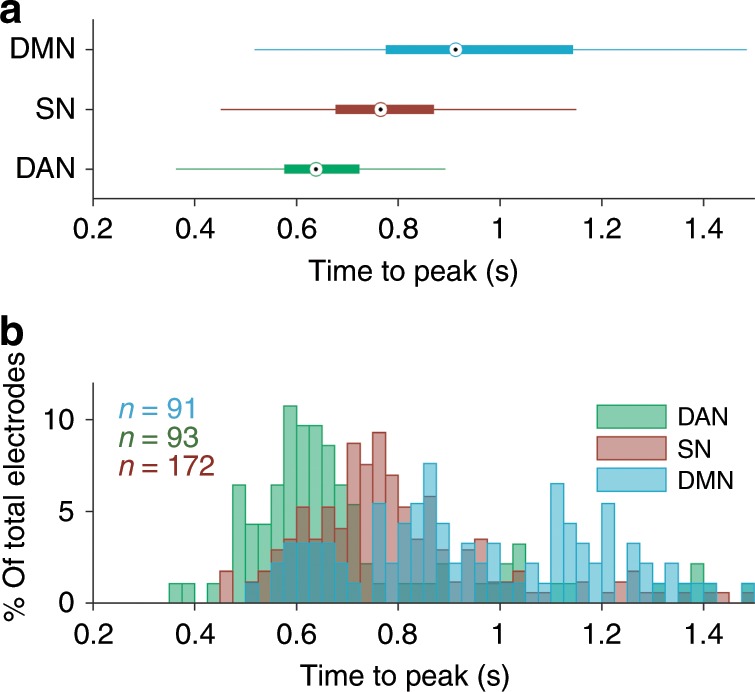
Time-to-peak (TTP) of task-evoked HFB responses varies among networks. **a** Box plots showing TTP for all task-responsive electrodes within each network (based on HFB increase for DAN and SN and HFB decrease for DMN). The central point indicates median, box bounds indicate 25th and 75th percentiles, and whiskers indicate most extreme data points not considered to be outliers. **b** Histogram of the distribution of TTP values for all electrodes.

We next sought to determine whether there was evidence for distinct timing of task-evoked HFB responses among the DAN, SN, and DMN. We computed the time-to-peak (TTP) of HFB response for each significant, task-responsive electrode (positive peak for DAN and SN, negative peak for DMN) during target trials with correct behavioral performance (withheld button press). We found that there was a significant interaction between TTP and electrode network assignment (*F*_1,357_ = 18.1, *p* = 2.6 × 10^−5^, *F* test on linear mixed effects model). The TTP was earliest in the DAN, intermediate in the SN, and latest in the DMN (Fig. 3a, b). Direct comparisons between network pairs revealed that DAN was significantly earlier than SN (*F*_1,265_ = 10.9, *p* = 0.001, *F* test on linear mixed effects model), DAN was significantly earlier than DMN (*F*_1,163 _= 50.9, *p* = 3.0 × 10^−11^, *F* test on linear mixed effects model), and SN was significantly earlier than DMN (*F*_1,264_ = 31.5, *p* = 5.0 × 10^−8^, *F* test on linear mixed effects model). Similar temporal profiles were seen during independent trials where subjects incorrectly responded to target stimuli (commission error trials) (Supplementary Fig. 4).

### Network-specific HFB profiles and behavioral errors

**Fig. 4:**
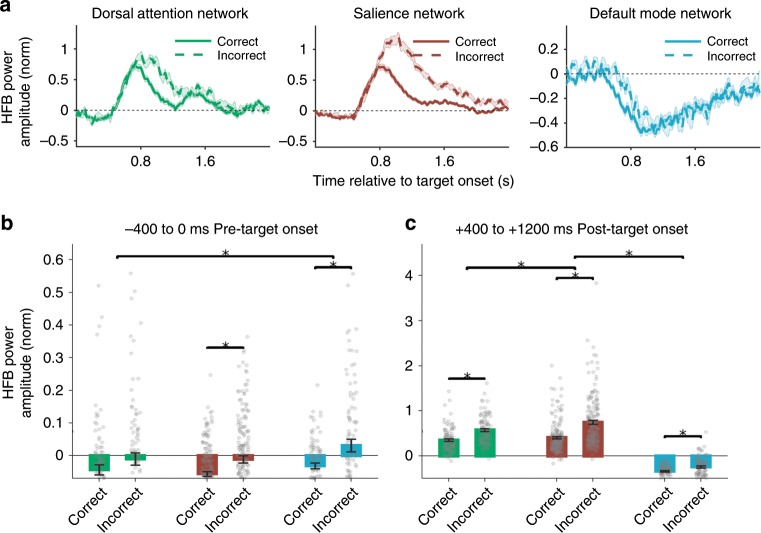
Network-specific profiles of task-evoked HFB activity during and preceding behavioral errors. **a** High-frequency broadband responses during the presentation of mountain (target) stimuli, split into trials with correct and incorrect behavioral responses. Time courses show the grand average of the trial-wise means within all task-responsive electrodes (*p* < 0.05, cluster-based permutation test, corrected for multiple comparisons within networks within subjects). **b** Mean HFB power amplitude within the − 400 to 0 ms window relative to target onset. **c** Mean HFB power amplitude within the + 400 to + 1200 ms window relative to target onset. In **b** and **c**, bars show the mean of normalized HFB within all task-responsive electrodes (averaged across trials) within a given network, and error bars indicate standard error of the mean across electrodes. Upper lines and asterisks indicate significant effects (*p* < 0.05, Satterthwaite’s approximation) based on *F* tests of linear mixed model analyses (see Methods). Error bars indicate standard error of the mean.

Beyond differences among networks in response timing, we next aimed to uncover how the dynamics of electrophysiological activity within the DAN, SN, and DMN might covary with lapses of attention (defined as commission errors), as has been suggested in previous fMRI studies^18,20,42^. Grand averages across all task-responsive electrodes indicated that, for target trials with both correct and incorrect behavioral responses, functionally localized DAN and SN regions showed increased HFB, whereas DMN regions showed decreased HFB (Fig. 4a). We tested whether HFB activity prior to, as well as during, trials differed as a function of behavioral response within and between networks.

In the −400 to 0 ms window prior to target trial onset, HFB power was significantly different between correct and incorrect trials within the SN (*F*_1,317_ = 15.6, *p* = 9.7×10^−5^, *F* test on linear mixed effects model) and DMN (*F*_1,184_ = 8.56, *p* = 0.004, *F* test on linear mixed effects model) but not within the DAN (*F*_1,167_ =  2.4, *p* = 0.12, *F* test on linear mixed effects model). In both the SN and DMN, these effects were driven by greater pre-target HFB power prior to incorrect relative to correct trials (Fig. 4b). The interaction between network and behavioral response for pre-target HFB power amplitudes was significant for DAN versus DMN (*F*_1,179_ = 4.4, *p* = 0.038, *F* test on linear mixed effects model) but not for DAN versus SN (*F*_1,265_ = 0.24, *p* = 0.62, *F* test on linear mixed effects model) or SN versus DMN (*F*_1,263_ = 0, *p* = 1.0, *F* test on linear mixed effects model). These findings suggest that increased HFB power in the DMN and SN signify precursors to lapses of attention (and that DAN activity shows a similar trend).

In the +400 to +1200 ms window following target trial onset, HFB power was significantly different between correct and incorrect trials within the DAN (*F*_1,167 _= 31.9, *p* = 6.9 × 10^−8^, *F* test on linear mixed effects model), SN (*F*_1,325 _= 58.3, *p* = 2.52 × 10^−13^, *F* test on linear mixed effects model), and DMN (*F*_1,164 _= 16.6, *p* = 7.0 × 10^−5^, *F* test on linear mixed effects model). In the DAN and SN, these effects were driven by greater post-target HFB power during incorrect relative to correct trials, whereas within the DMN, these effects were driven by lower post-target HFB power (i.e., a greater decrease) during correct relative to incorrect trials (Fig. 4c). The interaction between network and behavioral response for post-target HFB power amplitudes was significant for SN versus DAN (*F*_1,251_ = 13.38, *p* = 0.0003, *F* test on linear mixed effects model) and SN versus DMN (*F*_1,260_ = 8.03, *p* = 0.005, *F* test on linear mixed effects model) but not for DAN versus DMN (*F*_1,184.61 _= 2.54, *p* = 0.11, *F* test on linear mixed effects model). Thus, the SN had the strongest increase in HFB power during incorrect relative to correct trials relative to DAN and DMN.

### Behavioral significance of lagged inter-network antagonism

**Fig. 5:**
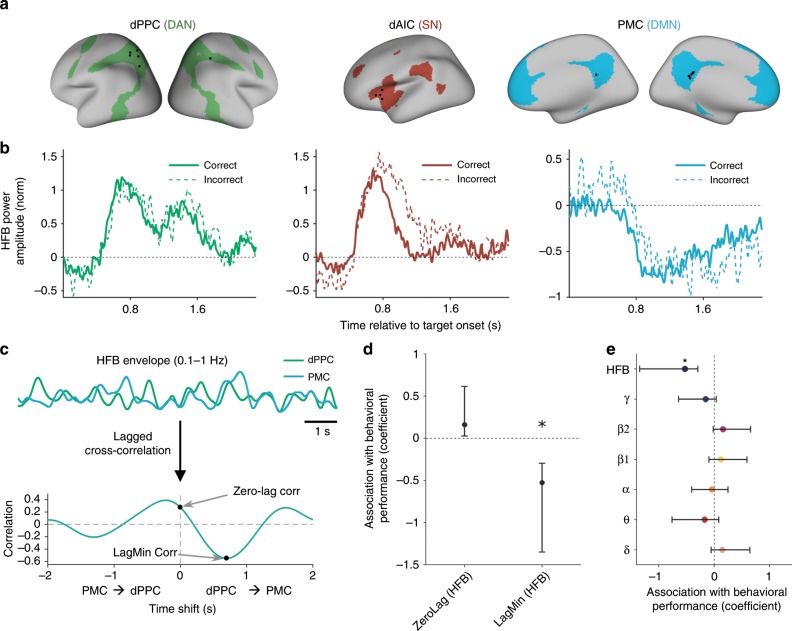
Relationship between behavioral performance and inter-electrode coupling of key DAN, SN, and DMN nodes. **a** Locations of peak-responsive dPPC, dAIC, and PMC electrodes (in fsaverage space) within cohorts of subjects with simultaneous dPPC-PMC (*n* = 6) and dAIC-PMC (*n* = 4) coverage. **b** Grand average HFB responses during GradCPT correct omission and commission error trials for the electrodes shown in **a**) (cf. Fig. 4a). **c** Illustration of how inter-electrode coupling was calculated from continuous HFB 0.1–1 Hz filtered time series. Using an example 20-sec time series for illustration purposes (top), a lagged cross-correlation was performed (shifting dPPC relative to PMC and vice versa). (Bottom) The zero-lag correlation was taken as the value with no time shift, whereas the lag-minimum correlation was taken as the minimum value across the time shifts. In the main analysis, these metrics were calculated based on whole runs (typically 6 min long). **d** Coefficients from a linear mixed model with d′ (behavioral performance) as dependent variable and with fixed factors including lag-minimum and zero-lag 0.1–1 Hz HFB correlation between dPPC and PMC (and including subject modeled as random factor). **e** Coefficients as in **d** for lag-minimum correlations in separate linear mixed effects models that were each constructed from power amplitudes of distinct frequency bands. Error bars indicate upper and lower 95% confidence intervals. Asterisks indicate significant effects (*p* <0.05, Satterthwaite’s approximation, *F* test on linear mixed effects model).

As our time-resolved iEEG analyses had revealed shifted (i.e., non-zero-lag) task-evoked HFB increases and decreases among networks, we next aimed to determine whether coordinated inter-network activity lags were behaviorally significant (i.e., varied with task performance across runs). To perform this analysis at the group level, it was important to ensure that the inter-network region pairs that potentially relate to behavior were anatomically matched between subjects. We thus focused on subjects with simultaneous task-responsive sites across key region-of-interests (ROIs) where electrode coverage was available within the DMN, DAN, and SN (Fig. 1c–f): (1) PMC within the DMN;^6^ (2) dPPC, including superior parietal lobule (SPL) and intraparietal sulcus (IPS), within the DAN^2^; and (3) dAIC within the SN (Fig. 5a)^4^. In resulting cohorts of six subjects with dPPC-PMC and four subjects with dAIC-PMC coverage, the peak-responsive electrodes within PMC, dPPC, and dAIC, respectively, showed task-evoked HFB activity profiles that closely resembled the grand average patterns that we had found for the whole DMN, DAN, and SN (Fig. 5b; cf. Fig. 4a).

We hypothesized that time-shifted, but not zero-lag, negative inter-electrode coupling between PMC and dPPC/dAIC would reflect a subject’s level of overall sustained attention that varied across GradCPT runs. We defined behavioral performance within each run by the measure of sensitivity (*d*′), which is based on the accuracy of task performance (accounting for both hits and false alarms)^43,44^. To assess the relationship with sustained attention performance (*d*′), we estimated the coordination of dPPC-PMC and dAIC-PMC activity across each task run using continuous HFB envelope fluctuations. Based on previous work indicating that coupled HFB power fluctuations in the 0.1–1 Hz resemble patterns of BOLD functional connectivity^31–34^, we expected that behaviorally significant time-lagged interactions would be most apparent in minimally filtered HFB signals. We computed both zero-lag correlations and lag-minimum HFB correlations, defined as the maximum negative correlation between regions for time series that could be shifted from −2 to +2 s (Fig. 5c).

We found that *d*′ was significantly associated with dPPC-PMC lag-minimum correlation (*F*_1,32 _= 13.3, *p* = 0.0009), but not with zero-lag correlation (*F*_1,32 _= 1.24, *p* = 0.27), across 35 task runs within the cohort of 6 dPPC-PMC subjects for the 0.1–1 Hz HFB range (*F* tests conducted on a linear mixed effects model with separate fixed factors for lag-minimum and zero-lag correlation). Specifically, the coefficients signified that greater dPPC-PMC lag-minimum, but not zero-lag, HFB correlation was associated with better sustained attention (higher *d*′) (Fig. 5d). The negative association between 0.1 and 1 Hz lag-minimum dPPC-PMC correlation and behavior was seen for HFB but not for lower frequency ranges of iEEG power amplitudes (Fig. 5e). When using the unfiltered, rather than 0.1–1 Hz filtered, HFB envelope, the relationship between *d*′ and dPPC-PMC lag-minimum correlation was weaker but remained significant (*F*_1,32 _= 7.45, *p* = 0.01, *F* test on linear mixed effects model).

In contrast to the dPPC-PMC findings, for dAIC-PMC coupling, sustained attention (*d*′) was not significantly associated with inter-electrode lag-minimum correlation (*F*_1,20_ = 0.24, *p* = 0.63, *F* test on linear mixed effects model) or with zero-lag correlation (*F*_1,20_ = 1.21, *p* = 0.28, *F* test on linear mixed effects model) of the 0.1–1 Hz HFB envelope across 23 task runs within the cohort of four dAIC-PMC subjects. When using the unfiltered dAIC and PMC HFB envelopes, the relationship between sustained attention and lag-minimum correlation was stronger but was not significant (*F*_1,20_ = 3.79, *p* = 0.07, *F* test on linear mixed effects model). Thus, in summary, dPPC-PMC (DAN-DMN) lagged anticorrelation was consistently associated with variations in attentional performance across time scales of HFB activity, whereas dAIC-PMC (SN-DMN) lagged anticorrelation was less consistently associated with performance.

### Lagged antagonism is reduced during wakeful rest

We sought to further confirm that lagged inter-network antagonism exhibited behavioral relevance over-and-above the contribution of zero-lag coupling. We hypothesized that during wakeful rest,—a state that typically promotes introspective and internally oriented thoughts^8^— there would be a reduction in time-shifted DMN-DAN/SN negative coupling of the 0.1–1 Hz filtered HFB envelope. We therefore compared the inter-network electrode coupling between sessions of externally oriented task performance (GradCPT) versus wakeful rest for subjects with simultaneous coverage of task-responsive DMN-DAN (13 subjects, 301 electrode pairs) and DMN-SN (19 subjects, 573 electrode pairs) electrodes. We included the coupling measures of lag-minimum and zero-lag correlation as separate fixed effects within single models to account for potentially overlapping variance between these measures.

**Fig. 6:**
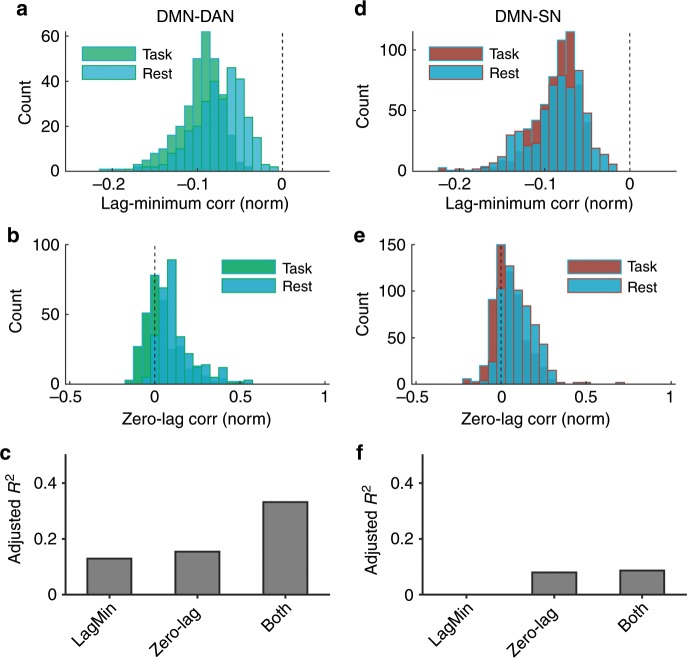
Lag-minimum and zero-lag correlation of 0.1–1 Hz HFB signals during task performance and rest. **a** Histogram of the distributions of Fisher-transformed lag-minimum correlation values for all task-responsive DMN–DAN electrode pairs. **b** Histogram of the distributions of Fisher-transformed zero-lag correlation values for all task-responsive DMN-DAN electrode pairs. **c** The adjusted *R*^2^ values for DMN–DAN linear mixed models with condition (task versus rest) as dependent variable and either lag-minimum correlation only, zero-lag correlation only, or both variables included as fixed effects. **d** Histogram of the distributions of Fisher-transformed lag-minimum correlation values for all task-responsive DMN–SN electrode pairs. **e** Histogram of the distributions of Fisher-transformed zero-lag correlation values for all task-responsive DMN–SN electrode pairs. **f** The adjusted *R*^2^ values for DMN–SN linear mixed models with condition (task versus rest) as dependent variable and either lag-minimum correlation only, zero-lag correlation only, or both variables included as fixed effects.

For DMN-DAN electrode pairs, task versus rest sessions exhibited significant differences, both in terms of lag-minimum (*F*_1,608 _= 158.2, *p* = 2.1 × 10^−32^, *F* test on linear mixed effects model) and zero-lag (*F*_1,507 _= 166.5, *p* = 3.8 × 10^−33^, *F* test on linear mixed effects model) coupling of HFB envelopes. During task relative to rest, lag-minimum correlation showed stronger anticorrelation of DMN-DAN electrode pairs (Fig. 6a), and zero-lag correlation was decreased in magnitude (Fig. 6b). The two coupling measures each explained unique variance in DMN-DAN differences between task and rest; an adjusted *R*^2^ value of 0.33 was obtained when accounting for both measures, whereas 0.15 (zero-lag) and 0.13 (lag-minimum) values were obtained when accounting for single measures each alone (Fig. 6c).

For DMN-SN electrode pairs, task versus rest sessions also exhibited significant differences, both in terms of lag-minimum (*F*_1,1164_ = 9.4, *p* = 0.002, *F* test on linear mixed effects model) and zero-lag (*F*_1,1164 _= 112.7, *p* = 3.4 × 10^−25^, *F* test on linear mixed effects model) coupling of 0.1–1 Hz HFB power amplitudes. However, during task relative to rest, lag-minimum correlation showed similar distributions across electrode pairs (Fig. 6d), whereas zero-lag correlation was decreased in magnitude (Fig. 6e). The two coupling measures did not explain unique variance in DMN-SN differences between task and rest: an adjusted *R*^2^ value of 0.087 was obtained when accounting for both measures, whereas 0.080 (zero-lag) and 0 (lag-minimum) values were obtained when accounting for single measures each alone (Fig. 6f). Thus, when accounting for lagged activity, there was improved distinction between task and rest conditions for DMN-DAN but not DMN-SN electrode pairs.

## Discussion

Here, using recordings from electrodes implanted directly in the human brain, we characterized the behavioral relevance of electrophysiological dynamics within and between large-scale antagonistic brain networks (DMN versus DAN and SN). We found that task-evoked responses within these networks during the presentation of behaviorally relevant external stimuli were best characterized by activity within the HFB (70–170 Hz) range. The HFB responses peaked fastest in the DAN, were at intermediate speed in the SN, and were slowest in the DMN. Relatively increased HFB power within the DMN and SN signified upcoming lapses of attention (behavioral errors), and the occurrence of behavioral errors was associated with dissociable HFB profiles among the three networks (with SN showing a most amplified increase during errors). Supporting the functional importance of temporal delays between antagonistic networks, we found that greater lagged, but not zero-lag, anticorrelated coupling between dPPC (a DAN region) and PMC (a DAN region) activity was associated with better sustained attention across repeated sessions of continuous task performance. Furthermore, lagged, anticorrelated coupling between the DMN and DAN was reduced during wakeful rest (a state marked by internally oriented thought) relative to externally oriented task performance. These findings underscore the behavioral relevance of previously unrecognized, temporally delayed coordination between antagonistic brain networks.

Antagonistic patterns of task-evoked DMN-DAN/SN activity have been found in functional neuroimaging studies across an extensive variety of task conditions involving both stimulus-driven and goal-oriented attention^45,46^. States of lapsing attention and mind-wandering, which are largely incompatible with sustained, externally oriented attention, have been associated with increased DMN activation, or a lack of DMN suppression^17,19,20^. It has thus been proposed that anticorrelated networks may continuously compete with one another for control of shared computational resources^47,48^.

Building on this framework, our finding that task-evoked DAN activations precede SN activations, and that both precede DMN deactivations, may point toward a causal chain of events that is required for successful deployment of stimulus-driven and goal-oriented attention. During a baseline (or low cognitive demand) state, the DMN maintains control over computational resources (e.g., for imagery associated with internally oriented cognition). When behaviorally relevant sensory information is successfully transferred to DAN/SN regions, those regions gain control over resources that the DMN previously had access to, and subsequently the DMN is actively suppressed. During lapses of attention, pre-existing neural activity is in a state of high DMN and SN engagement, and incoming behaviorally relevant stimuli may not be processed efficiently. Such a pre-trial state, potentially associated with mind-wandering, appears to involve a lack of opposing activity between networks (DMN versus SN) that otherwise function antagonistically when attentional resources are successfully deployed. Delayed and attenuated processing of sensory input would result^49^ and would manifest as behavioral errors.

An important caveat is that our finding of a systematic temporal order of electrophysiological responses does not necessarily imply causal interactions among the DAN, SN, and DMN, and further study on directional relationships will be needed. Our results may, however, impose important constraints on models of network dynamics, as they leave open the possibility that task-evoked DAN and SN activations could influence DMN deactivations, whereas they argue that DMN deactivations are unlikely to influence DAN and SN activations. The relatively late suppression of the DMN relative to activation in other association networks suggest a temporal hierarchy that may accord well with findings that situate DMN regions as those with longest connectivity paths and furthest geodesic distance from primary sensory regions^50^. Moreover, our findings accord with other iEEG studies showing relatively late DMN task-evoked responses^29,51^ as well as with work suggesting that temporal receptive windows are prolonged within DMN compared with other higher-order association regions^52,53^.

The dynamics of anticorrelated activity in relation to task performance have previously been studied largely with group-level fMRI, focused on zero-lag interactions of slow hemodynamic signals. States of greater DMN-DAN/SN anticorrelation have been associated with greater vigilance and behavioral stability^15,16,54,55^. Across individuals, greater baseline BOLD anticorrelation has been associated with lesser response time variability^56^, fluid intelligence^57^, and greater working memory capacity^58^—all of which are behavioral measures that may rely on sustained attention. Moreover, attenuated anticorrelation has been found in clinical conditions involving attentional dysfunction^59,60^ as well as in cognitive decline with aging^58,61^.

Our findings extend those results in multiple ways: first, we provide critical neurophysiological validation for DMN-DAN/SN antagonistic activity profiles during task-related sustained attention at the level of functionally localized neuronal populations. Second, we show that electrophysiological activity within and between these networks is associated with momentary changes in sustained attention. Third, and most importantly, we show that behaviorally relevant, antagonistic activity involves inter-network lags of up to hundreds of milliseconds that are too short for fMRI to detect. Our iEEG analyses may have been sensitive to behaviorally relevant, time-resolved interactions that would not be detectable with current human neuroimaging methods. Despite the low temporal resolution of functional neuroimaging, inter-network directional and lagged interactions have long been of interest in task and resting states^62^. As fMRI studies contend with regional heterogeneity in blood flow dynamics^63^, it remains an open question whether advances with accelerated neuroimaging will allow detection of temporally ordered, anticorrelated DMN-DAN/SN activity and its variation over time.

In the functional neuroimaging literature, the antagonistic relationship between the DMN and DAN—potentially highlighting a competition between internally- and externally oriented attention—has received intense focus and scrutiny. Though typically lesser emphasized, the SN, also shows negatively correlated BOLD activity with the DMN^7,64,65^. Functional neuroimaging evidence indicates that the SN and DAN have dissociable roles in externally oriented attention. The DAN shows domain-general activation (in tandem with DMN deactivation) during various conditions involving goal-oriented attention^2^. The SN shows activation during detection of salient external stimuli^66^ and during behavioral errors in fMRI^67^, iEEG^68^, and single-unit recordings from key SN nodes^69^. It has been proposed that the SN, and the dAIC in particular, causally facilitates switching between other networks (including DMN and DAN) to reorient attention during salient event detection^3,5^. In partial agreement, a recent application of dynamic causal modeling to resting state fMRI data suggested that the SN and DAN exert intrinsic inhibitory influences on the DMN^70^. Our iEEG results extend these frameworks and confirm the presence of electrophysiological DMN-SN and DMN-DAN antagonistic interactions during continuous task performance. The temporal profiles of task-evoked activity that we identified are broadly compatible with the possibility that the SN could act as a switch between the DAN and DMN.

We found strong evidence for dissociable electrophysiological activity in the DAN and SN. First, the task-evoked SN responses peaked several hundreds of milliseconds later than DAN responses. Second, compared with the DAN (and DMN), the SN was more likely to show increased HFB activation during incorrect relative to correct behavioral responses. Third, DMN-DAN, compared with DMN-SN lagged anticorrelation, was more strongly associated with attentional performance. Fourth, DMN-DAN but not DMN-SN lagged anticorrelation was reduced during wakeful rest compared with continuous task performance. Interestingly, fMRI evidence indicates that the SN may flexibly couple with either the DAN or DMN based on task conditions^71^. Though our results here were based on an externally oriented continuous performance task, future iEEG studies exploring distinct cognitive processes may provide further insight into context-dependent temporal dynamics of DAN, DMN, and SN interactions.

The fMRI-based finding of anticorrelated networks during wakeful rest has led to the notion that there could be an intrinsic, state-independent, antagonistic relationship between the DMN and other networks^7^. Under this framework, the brain may continuously shift between states that draw, respectively, from internally- and externally oriented sources of information^8,12^. However, the concept has remained controversial, in large part due to technical limitations of fMRI^10^. Infraslow resting state BOLD anticorrelations become introduced into data following preprocessing with global signal regression^21^, but anticorrelations have also been detected in the absence of global signal regression and with alternative noise-correction strategies^72,73^. Though anticorrelation of infraslow inter-network activity has been recovered in computational models^74^, electrophysiological DMN-DAN/SN anticorrelations are not typically reported in non-invasive M/EEG^75–77^. However, using 3–6 min resting state iEEG recordings, Keller et al.^32^ showed that a subset of region pairs with resting BOLD anticorrelations exhibited anticorrelated 0.1–1 Hz HFB activity (of smaller magnitude compared to those found in BOLD data), especially when global signal regression was applied to iEEG data.

In our work, we did not apply global signal regression, and there was an absence of zero-lag resting state anticorrelation in the 0.1–1 Hz HFB range for most of the DMN-DAN and DMN-SN electrode pairs investigated. One possible explanation for this stems from our finding of coordinated inter-network delays, which could suggest that intrinsic negative interactions between networks may be better detected when accounting for lags on the order of hundreds of milliseconds. It is possible that the hemodynamic response “blurs” this inter-network temporal lag over time, resulting in zero-lag negative BOLD correlations. Further study of the spontaneous electrophysiological dynamics of inter-network interactions, potentially combined with BOLD imaging and/or hemodynamic response modeling (see ref. ^78^), is needed to test this hypothesis.

In conclusion, our findings establish a behavioral significance of systematic temporal lags underlying the coordination of activity between antagonistic brain networks. This knowledge is critical for the interpretation of task and resting state functional neuroimaging studies and for understanding the basis of changes in inter-network relationships in health, aging, and disease. The temporally ordered inter-network interactions identified here point toward the possible capacity for causal influences, a topic that requires further study with neuromodulatory techniques such as direct brain stimulation.

## Methods

### Subjects

Data from 31 human subjects (S1–31) who were undergoing neurosurgical treatment for refractory focal epilepsy were included in analyses reported here. Data from S1–10 were collected at Stanford University Medical Center, whereas data from S11–31 were collected at Beijing Tian Tan Hospital (see Supplementary Table 1 for full demographic and other details). Subjects were implanted with intracranial electrodes that either were depth electrodes placed stereotactically within one or both hemispheres (29 subjects) or subdural electrodes arranged in grid and strip configurations over one hemisphere (two subjects). Electrode placement was decided based on clinical evaluation for resective surgery. Intracranial electrode monitoring took place over the course of multiple days. Subjects at all experiment sites provided verbal and written informed consent to participate in research. For procedures at Stanford, the Stanford Institutional Review Board approved all procedures described herein. For procedures at Beijing, the Medical Ethics Committee of Beijing Tian Tan Hospital approved all procedures.

Subjects included in analyses presented here were selected from a cohort of 46 patients who participated in the cognitive task procedures herein. Exclusion criteria were as follows: (1) poor behavioral performance (criteria described below) that could indicate lack of compliance with task instructions or inability to perform the task successfully (*n* = 12); (2) major structural brain abnormalities that impeded MRI-based cortical surface reconstruction, including encephalomalacia and damage from prior resections (*n* = 3).

### Intracranial EEG data acquisition

iEEG recordings were performed at bedside of the subject’s private clinical suite. For Stanford patients, data were recorded with a Nihon Kohden (Tokyo, Japan) clinical monitoring system using a sampling rate of 1000 Hz and a bandpass filter of 1.6–300 Hz. For Beijing patients, data were recorded with a Nihon Kohden system using a sampling rate of 1000 Hz (bandpass filter of 0.08–300 Hz) or 2000 Hz (bandpass filter of 0.08–600 Hz). See Supplementary Table 2 for recording parameters. For Stanford patients, depth electrode contacts (Ad-Tech Medical Instrument Corporation, Oak Creek, WI, USA) were cylindrically shaped (0.86 mm diameter, 2.29 mm height) with inter-electrode spacing of 5–10 mm. For subdural electrodes, contacts were circle-shaped with diameter of 2.3 mm in the exposed area of recording and inter-electrode spacing of 5–10 mm. For Beijing patients, depth electrode contacts (HKHS Healthcare, Beijing, China) had a contact length of 2 mm, diameter of 0.8 mm, and inter-electrode spacing of 1.5 mm. During recording, the iEEG signals were referenced to the most electrographically silent channel outside of the seizure focus. The total number of unique electrode sites within subjects ranged from 48 to 210 (Supplementary Table 2).

### Continuous performance task sessions

The GradCPT^18^ was administered in multiple runs (range: 4–8) for each patient, with each run lasting 2–8 min (see Supplementary Table 2 for the number of runs obtained and total task duration per subject). The number of runs obtained within each patient depended on time available for research testing in the clinical environment, which varied across patients. The task was administered at bedside, in multiple sessions when necessary, via a laptop (running Windows 10 Pro and Windows 8.1, respectively, in Stanford and Beijing) with its screen positioned ~70 cm from the patients’ eyes at chest level. Stimuli were presented using Psychophysics Toolbox^79^ in Matlab R2016b (MathWorks, Natick MA, USA). An RTBox device^80^ was used to send transistor–transistor logic pulses to an empty channel on the EEG montage to mark the onset times of each stimulus.

During task performance, grayscale visual images of either city or mountain scenes appeared within round frames (with white background) and gradually transitioned from one to another for the duration of the task. Each transition lasted 800 ms. Using linear pixel-by-pixel interpolation within each trial, image coherence began to gradually increase from time zero (minimum coherence) until 400 ms (maximum coherence) before gradually decreasing back to minimum coherence (at 800 ms). In the majority of runs, scenes were presented randomly with 10% mountain and 90% city, but the same scene could not repeat on consecutive trials. In eight subjects (S11–18), half of the runs were performed with 25% mountain and 75% city rates; these runs were included in all analyses except for those of inter-run variability in task performance, owing to potential changes in task difficulty). For both cities and mountains, there were 10 unique images. The presented order of these unique images was randomized on each iteration of the task to mitigate learning specific sequences. Subjects were instructed to press the space bar on the laptop upon noticing each city appearing but to withhold response when noticing a mountain appearing. Subjects were asked to perform their best and to keep going when they noticed themselves making an error. Each task session began with a 20 s baseline period in which the patient was instructed to fixate on a blurred mask stimulus (same size as scene stimuli) and get ready to begin. Subjects performed with their dominant hand, except in occasional situations where there was discomfort of the dominant hand.

### Resting state recordings

Resting state runs were obtained and analyzed within 19 patients included in our final cohort. Prior to resting state recordings, subjects were asked to relax and not think of anything in particular, whereas either keeping eyes closed or open. In select resting state runs, subjects were instructed to fixate on a central cross on the laptop screen.

### MRI acquisition

In a pre-operative MRI session, all subjects underwent structural MRI (T1-weighted). In addition, a computed tomography (CT) scan was obtained following electrode implantation, which was used for anatomical localization of electrode contacts. For Stanford patients, neuroimaging was performed at Stanford Hospital on a 3.0 Tesla GE 750 MR system equipped with an eight-channel receive only head coil (8HRBrain). In Beijing, neuroimaging was performed at Beijing Dongzhimen Hospital on 3.0 Tesla Siemens MAGNETOM Verio system with a 32-channel head coil. For T1 scans at Stanford, the parameters were 256 × 256 matrix, 160 slices, 0.94 × 0.94 × 1.00 mm voxels, 240 mm field of view, 13 degree flip angle, 9.63 ms repetition time, and 3.88 ms echo time. For T1 scans in Beijing, an MPRAGE sequence was acquired with parameters 256 × 256 matrix, 176 slices, 1.00 × 1.00 × 1.00 mm voxels, 9 degree flip angle, 1900 ms repetition time, and 2.53 ms echo time.

### Anatomical localization of electrode contacts

We used the iElvis pipeline^81^ for anatomical localization of electrode contacts. First, we processed and reconstructed the T1 scan using Freesurfer v6.0.0 (*recon-all* command)^82^. We then aligned the post-implant CT image to the pre-implant T1 scan using a rigid transformation (six degrees-of-freedom, affine mapping), and we inspected the quality of the registration. Using BioImage Suite^83^, we manually labeled each electrode location on the T1-registered CT image. For subdural electrode cases, we then projected the electrode coordinates to the leptomeningeal surface and applied correction for post-implant brain shift, using previously described methods^84^. In stereotactical EEG cases, minimal post-implant brain shift is expected, and thus no further adjustment was made. The electrode coordinates obtained from these approaches were used for visualization and to assign electrodes to anatomical regions and networks of interest.

### Anatomical classification of electrode contacts

We assigned each electrode to membership within the DMN, DAN, SN, or none of these three networks, based on alignment with the Yeo population-level standard atlas of seven cortical networks^37^. To do so, we registered the Yeo atlas from fsaverage space to individual cortical surfaces such that each vertex on the pial surface was classified within one of the seven networks^81^. For subdural electrode contacts, network assignment was based on the brain shift-corrected vertices. For depth electrodes, as coordinates were in volume (rather than surface) space, we assigned each electrode to the nearest cortical and white matter vertex. Depth electrodes were considered to be within cortex and were assigned to a cortical network only if the following conditions were met: the squared distance to the nearest cortical vertex was shorter than that (a) to the nearest white matter vertex, (b) between the nearest cortical and white matter vertices, and (c) was <4 mm.

We additionally conducted ROI based analyses of the dPPC, PMC, and dorsal anterior insula (dAIC) owing to their well-described memberships within the DAN, DMN, and SN, respectively (see Analyses of Inter-Electrode Coupling and Task Performance). We classified electrode contacts as being within the dPPC, PMC, or dAIC based on individual-level anatomy reviewed on 3D T1 volumes and cortical surface reconstructions.

As both the superior parietal lobule (SPL) and intraparietal sulcus (IPS) have been linked to the DAN^2,85^, we considered these adjacent areas within the parietal lobe as a single ROI, which we term dPPC. The IPS part of the dPPC was considered as the sulcus which runs along the anterior-posterior axis within lateral parietal cortex, approximately from the post-central sulcus to the transverse occipital sulcus. The SPL part of the dPPC (Brodmann area (BA) 7) included the parietal cortex regions that are medial to the IPS, extending in the anterior-posterior axis from the post-central sulcus to the parieto-occipital sulcus.

We defined the PMC as in previous work^34,86,87^. The PMC included areas posterior to the post-central sulcus within the posterior cingulate cortex (within BA 23a and 23b), retrosplenial cortex (BA 29/30), and medial parietal cortex/precuneus (BA 31 and 7 m). These areas were bounded by the marginal branch of the cingulate sulcus (dorsally/anteriorly) and by the parieto-occipital sulcus (posteriorly).

The dAIC was defined based on boundaries and landmarks defined previously^88,89^ to demarcate areas that corresponded largely to the agranular anterior insular zone^90^. This included the accessory gyrus of the insula, and/or portions of the anterior, middle and posterior short gyri of the insula that were superior to the inferior-most point of the short insular sulcus. These dorsal anterior subregions of the insula have been consistently linked with the salience, or cingulo-opercular, network^4,91^.

### Intracranial EEG: data preprocessing

Data from iEEG recordings were preprocessed similarly for task and rest runs using a pipeline consistent with previous work^33^. The procedures drew from tools in the Matlab-based LBCN preprocessing pipeline (https://github.com/LBCN-Stanford/Preprocessing_pipeline), SPM12^92^, and Fieldtrip^93^. For task runs, the recording was first cropped to retain data only within the pre-task baseline and task performance periods. Notch filtering was performed to attenuate power-line noise (zero-phase, third order, butterworth filter with band-stop between 57–63, 117–123, and 177–183 Hz for data from Stanford, and band-stop between 47 and 53, 97–103, and 147–153 Hz for data from China). We then re-referenced the signal from each channel to the common average signal across all channels, with the following channel types excluded from the common average: those that (a) showed pathological activity during clinical monitoring (as noted by a neurologist); (b) were manually labeled as clear outliers on power spectra plots of all channels; (c) had a variance greater or lesser than five times the median variance across all channels; or (d) had greater than three times the median number of spikes across all channels, with spikes defined as 100 μV changes between successive samples. We then performed time-frequency decomposition using a Morlet wavelet transform with frequencies of interest log-spaced between 1 and 170 Hz (38 total values). To normalize the distributions of power amplitude estimates, for each frequency of interest, we rescaled each time sample by the log ratio of the whole run’s power amplitude time series. This rescaling step accounted for the band-specific 1/*f* decline of the power spectrum^94^. Subsequently, we performed averaging of power amplitude estimates within seven frequency bands, including *δ* (1–3 Hz), *θ* (4–7 Hz)*, α* (8–12 Hz)*, β1* (13–29 Hz)*, β2* (30–39 Hz)*, γ* (40–70 Hz), and HFB (70–170 Hz). We then visually inspected the HFB time series in each run, and we excluded electrodes that showed irregular, spikey or pathological activity (that may have been otherwise missed in our inspection/exclusion prior to time-frequency decomposition).

### Behavioral analysis

Because of the fast pace of the GradCPT and the overlap of stimuli across adjacent trials, key presses were assigned to trials using a previously described iterative algorithm^18,19^. Presses were assigned relative to the beginning of each image transition. For trials in which the reaction time (RT) was highly deviant (before 70% image coherence for current trial, or after 40% coherence for the following trial), the following criteria were used for trial assignment: (1) If the previous or current trial had no response, the press was assigned to the trial in which the response occurred; (2) If both adjacent trials had no response, the press was assigned to the trial closest in time (excluding cases where the trial was a mountain image). (3) If multiple presses could be assigned to a given trial (based on (1) and (2)), the fastest RT was assigned to that trial. Based on these trial assignments, we computed the rates of omission errors (withheld button presses to non-target city scenes) and commission errors (button presses to target mountain scenes) within each run. Subjects were included in analyses only if they had performed at least four GradCPT runs that each had omission and commission error rates < 15% and 60%, respectively (i.e., approximately three times greater than the average error rates reported in other healthy and patient populations^18,36^).

We used sensitivity (*d*′) as a measure of overall task performance, based on signal detection theory^95^, within each session:

$$d\prime = Z\left( {hit\,rate} \right) - Z\left( {false\,alarm\,rate} \right)$$

where *Z*(*p*) is the inverse of the cumulative distribution function of the Gaussian distribution. Thus, the higher the *d*′ value, the higher the overall accuracy of behavioral performance (based on responses to both cities and mountains). Evidence indicates that *d*′, based on GradCPT performance, is a generalizable measure of sustained attention^43,44^.

### Functional localization of task-responsive iEEG electrodes

After assigning electrodes to membership within networks, we retained for analysis only those electrodes that were anatomically within the DMN, DAN, or SN. To functionally localize all task-responsive sites within these networks, we screened electrodes for evoked HFB power amplitude during correct omissions (withheld behavioral responses) to rare, target trials (mountain scenes) relative to correct commissions (behavioral responses) to frequent, city trials in the GradCPT. Based on replicated findings from previous fMRI studies^18,36^, and on the known association between BOLD activity and electrophysiological HFB activity^25,41,96,97^, we expected that HFB power would show an increase in DAN and SN sites as well as a decrease in DMN sites during correct omissions and commission errors.

For this analysis, we minimally smoothed the HFB power amplitude time course within each session using a 50-ms Gaussian window. We then extracted the HFB time series from windows surrounding each mountain trial, with each window starting at 800 ms prior to mountain scene onset (start of fade-in) and ending at 1600 ms after the onset. We excluded mountain trials that were preceded by other mountain trials. We also extracted time windows with the same boundaries around correct commission trials (city trials with button presses). For these correct commissions, we extracted only those that were both preceded and followed by other city trials (and both with correct responses) to avoid potential contamination with responses evoked by rare mountain scenes. Among these retained correct commission trials, we deleted a random subset of trials within each session such that the remaining subset included a total number of trials that matched the number of mountain (target) trials within the same run. For the purposes of this analysis, we refer to mountain and correct commission city trials as “target” and “baseline” trials.

To assess significance of HFB responses during target compared to baseline trials, we adopted a nonparametric cluster-based permutation test as implemented in Fieldtrip^38^ conducted separately for each network within each subject and accounting for multiple electrodes within each network. Combining trials across GradCPT runs within each subject, we performed independent samples *t* tests on normalized HFB power amplitude values to compare conditions, using data from each time point ranging from time zero to +1500 ms relative to trial onset (beginning of stimulus fading in). A two-tailed threshold of *p* = 0.05 was applied to the obtained t-values. Subsequently, adjacent samples exceeding the threshold were grouped together into clusters. The sum of t-values within each cluster was calculated for cluster-level statistics, and the maximum of those values was taken as the test statistic. These procedures were then repeated using the Monte Carlo method with 1000 randomizations of trials. Channels including observed clusters with a Monte Carlo significance probability less than 0.05 (two-tailed) were considered as significant. Based on this screening procedure, we retained for further analysis those SN and DAN sites that showed significant temporal clusters of increased HFB and those DMN sites that showed significant temporal clusters of decreased HFB.

### Multiple kernel learning analyses of distinct frequencies

To comprehensively assess the possible contributions of different frequency bands of activity to task-evoked iEEG responses, we performed a multiple kernel learning (MKL)-based analysis. The MKL approach is a machine learning-based method for feature selection that can be applied to classifying iEEG task conditions by including multiple frequency bands of activity as well as multiple electrodes in a single model^40^. We used MKL in the PRoNTo toolbox^98^ to classify correct omission (city) versus correct commission (mountain) trials.

For each subject, all electrodes that were anatomically identified as being within one of the networks of interest (DMN, DAN, SN) were included in the model. For each trial, power amplitudes from each electrode were extracted and averaged between time 0 to 1500 ms after trial onset for seven frequency bands (*δ, θ, α, β1, β2, γ*, HFB). As there were more correct commission than correct omission trials in each subject due to the nature of the task, we randomly subsampled from correct commission trials such that we obtained matched trial numbers across categories. Model features were defined as kernels, or pair-wise similarity matrices across the time series of all trials, which were constructed for each electrode and frequency band (i.e., the number of kernels per subjects was *m* × 7, with *m* being the number of electrodes). Each kernel was normalized and mean-centered to ensure that modeling was not influenced by the scale of each kernel.

We then applied MKL, using a support vector machine to define a decision boundary to discriminate between correct omission and correct commission trials. As in previous work^40^, model parameters were optimized to determine the decision boundary for each kernel, and decision boundaries were weighted by a parameter *d*_*m*_ to define a global decision boundary. We used a 10-fold cross-validation scheme: in each fold, training was performed on 90% of trials, and testing was performed on the 10% left out trials (with a different 10% left out on each fold). Model accuracy was obtained as the average balanced accuracy (average of class accuracies) across folds. During cross-validation, the soft-margin parameter, *C*, was optimized by considering values 0.01, 0.1, 1, 10, 100, and 1000. A nested cross-validation was performed where the value of *C* leading to highest model performance in the inner cross-validation was selected, and that *C* value was used to estimate performance in the outer cross-validation. To evaluate the contributions of different frequency bands of activity to model performance, for each fold we calculated the sum of *d*_*m*_ values across electrodes for each of the seven frequency bands. We then calculated the mean of those sums across the ten folds. We performed a repeated-measures ANOVA to test the hypothesis that distinct frequency bands would differ in their contributions to trial-type discrimination. We also performed post-hoc paired *t* tests (two-tailed) to test our hypothesis that HFB would have stronger contributions than other frequency bands (significance set at FDR-corrected *p* of 0.05).

### TTP analyses

We performed TTP estimation of HFB responses separately for correct omission versus commission errors trials and for all identified task-responsive electrodes in the DAN, SN and DMN. Within a time window ranging from +200 to +1500 ms after trial onset (i.e., beginning of mountain scene fade-in), we identified the maximum peak time point (for DAN and SN) or minimum peak time point (for DMN) for each electrode’s trial-wise mean HFB response. The +200 ms bound was selected a) to avoid possible contamination with responses to previous city stimuli, and b) based on prior iEEG findings suggesting that earlier peaks were unlikely to be plausible for DAN and DMN regions^29,34^. The +1500 ms bound was selected to limit possible contamination with responses to subsequent stimuli in the task. We used a linear mixed model to test whether electrodes within the DAN, SN, and DMN had distinct TTP distributions. Subject was entered as a random effect, network identity (DAN, SN, or DMN) was entered as a fixed effect, and TTP was entered as the dependent variable. We also performed three post-hoc tests that were based on similar linear mixed effects models but that had two rather than three network identities entered as the fixed effect (i.e., DAN and SN, DAN and DMN, DMN and SN). For these linear mixed models and those described below, we performed *F* tests on the coefficients with significance set at *p* < 0.05 (Satterthwaite’s approximation), two-tailed.

### Comparison of correct versus incorrect trials

We compared HFB activity across DAN, SN, and DMN electrodes prior to and during correct omission versus commission error trials. For each electrode, we computed the mean HFB power amplitude prior to target (mountain) onset in a −400 to 0 ms window (selected to capture the brain state immediately preceding the target) as well as a post-target window of +400 to +1200 ms (selected to capture the time period when the highest amplitude responses were expected, while minimizing contamination with responses during subsequent trials). For both the pre- and post-target HFB estimates (each separately), averaged across trials for each electrode, we used linear mixed effects models to test whether there were differences between correct omission versus commission error trials within the DMN, DAN and SN. For each network, subject was entered as a random effect, behavioral accuracy (correct omission versus commission error) was entered as a fixed effect, and HFB power amplitude (pre- or post-target) was entered as the dependent variable. Additionally, to test for interactions between network and behavioral accuracy, we performed linear mixed effects models (two-tailed) with subject entered as a random effect, network identity entered a fixed effect, and the difference in HFB power amplitude between commission errors and correct omissions (means subtracted between trial types) entered as the dependent variable.

### Analyses of inter-electrode coupling and task performance

To assess whether coupling between antagonistic networks varied with task performance across GradCPT runs, we focused on subjects who had simultaneous coverage of task-responsive electrodes within key inter-network node pairs that were anatomically matched across subjects. This included 6 subjects with dPPC-PMC coverage and 4 subjects with dAIC-PMC. Analyses were conducted using the peak-responsive electrodes within each ROI (as defined based on cluster-based permutation testing described above). To assess coupling between regions in continuous HFB power amplitudes, we applied a bandpass temporal filter (zero-phase, butterworth, 4th order) to the unsmoothed HFB envelope, retaining frequencies between 0.1 and 1 Hz^31–33^. We performed additional analyses of the HFB envelope, based on no filtering (minimally smoothed, as described above). We deleted the 20-second pre-task baseline window for these analyses. Inter-electrode coupling was assessed using two metrics: (1) Zero-lag correlation, and (2) Lag-minimum correlation: the minimum correlation (i.e., the greatest negative correlation) among cross-correlations between the electrodes’ time series for inter-electrode shifts ranging from −2 to 2 s. We then applied a Fisher *r*-to-*z* transformation to these values.

To compare inter-electrode coupling with run-to-run variability in behavioral performance (*d*′) across subjects, we first normalized the inter-electrode coupling and *d*′ values within subjects (i.e., for each run, we subtracted out the mean and then divided by the standard deviation of values across runs). We then performed linear mixed effect model analyses (two-tailed) with subject entered as a random effect, behavioral performance (*d*′) as dependent variable and inter-electrode coupling as fixed effects (zero-lag correlation and lag-minimum correlation as separate variables). Only GradCPT sessions that included 90% city and 10% mountain rate were included in these analyses so that task difficulty was matched across sessions.

### Analyses of inter-electrode coupling during task versus rest

We compared HFB inter-electrode coupling across task performance versus resting state for all subjects with simultaneous coverage of task-responsive DMN-DAN (13 subjects, 301 electrode pairs) and DMN-SN (19 subjects, 573 electrode pairs) electrodes and who had undergone one or two resting state runs (Supplementary Table 2). Within each run (task and rest), we filtered the continuous HFB power amplitudes to the 0.1–1 Hz range as described above. We deleted the 20-second pre-task baseline window for each task run and the first 20 s from each rest run. For each inter-network electrode pair, we computed inter-electrode coupling in two ways: (1) lag-minimum correlation, and (2) zero-lag correlation. We applied a Fisher *r*-to-*z* transformation to these values and then averaged the values across runs (when more than one run was available). For both DMN-DAN and DMN-SN electrode pairs, we then performed the following linear mixed effects model analyses (two-tailed): (1) condition (task versus rest) as dependent variable, lag-minimum correlation as fixed effect, and subject as random effect; (2) condition as dependent variable, zero-lag correlation as fixed effect, and subject as random effect; and (3) condition (task versus rest) as dependent variable, lag-minimum correlation and zero-lag correlation each as separate fixed effects, and subject as random effect.

### Reporting summary

Further information on research design is available in the Nature Research Reporting Summary linked to this article.

## Supplementary information

Supplementary Information

Reporting Summary

## Data Availability

The data that support the findings of this study are available from the corresponding author on reasonable request.

## Code availability

Code is publicly available for preprocessing of iEEG data (https://github.com/LBCN-Stanford/Preprocessing_pipeline) as in this study. The code for analyzing the data of this study are available from the corresponding author on reasonable request.
