# Supplementary material for: Electrophysiological dynamics of antagonistic brain networks reflect attentional fluctuations
Source: Nat Commun. 2020 Jan 16;11:325. doi: 10.1038/s41467-019-14166-2 (PMC6965628; doi:10.1038/s41467-019-14166-2)
Supplement: Supplementary file 1 — Supplementary Information [file 41467_2019_14166_MOESM1_ESM.pdf]

Supplementary Information

Electrophysiological dynamics of antagonistic brain networks reflect attentional fluctuations

Kucyi et al.

	Site	Subdural /Depth	iEEG sampling rate (Hz)	Age	Sex	L/R Handed	Epilepsy duration	Epileptic focus/activity
**S1**	Stanford	Subdural	1000	21	M	R	8 years	Right posterior temporal lobe
**S2**	Stanford	Depth	1000	36	M	R	26 years	Left occipital and medial temporal lobe
**S3**	Stanford	Depth	1000	31	M	R	7 years	Bilateral temporal lobe
**S4**	Stanford	Depth	1000	22	F	R	2 years	Right temporal pole
**S5**	Stanford	Subdural	1000	30	F	R	20 years	Left occipital and angular region
**S6**	Stanford	Depth	1000	19	F	R	5 years	Left lateral temporal lobe
**S7**	Stanford	Depth	1000	34	F	R	18 years	Left medial and lateral temporal lobe
**S8**	Stanford	Depth	1000	27	F	R	19 years	Left precentral gyrus
**S9**	Stanford	Depth	1000	23	M	R	4 years	Right medial temporal lobe
**S10**	Stanford	Depth	1000	49	M	R	23 years	Left medial posterior temporal lobe
**S11**	Beijing	Depth	2000	22	F	R	20 years	Right medial temporal lobe
**S12**	Beijing	Depth	1000	27	M	R	13 years	Right supramarginal gyrus and postcentral gyrus
**S13**	Beijing	Depth	2000	27	M	R	16 years	Left inferior parietal lobule
**S14**	Beijing	Depth	1000	37	M	R	3 years	Right orbitofrontal cortex
**S15**	Beijing	Depth	1000	19	F	R	6 years	Right medial temporal lobe
**S16**	Beijing	Depth	2000	26	M	R	2 years	Left medial temporal lobe, posterior cingulate and inferior parietal lobe
**S17**	Beijing	Depth	1000	33	M	R	33 years	Left lateral inferior temporal lobe
**S18**	Beijing	Depth	2000	19	F	R	3 years	Left orbitofrontal cortex
**S19**	Beijing	Depth	2000	19	F	L	3 years	Right lateral temporal lobe
**S20**	Beijing	Depth	1000	17	F	R	3 years	Right temporal pole
**S21**	Beijing	Depth	2000	18	F	R	9 years	Left medial temporal lobe
**S22**	Beijing	Depth	1000	27	F	R	26 years	Right superior frontal gyrus
**S23**	Beijing	Depth	2000	21	F	R	5 years	Right medial temporal lobe
**S24**	Beijing	Depth	1000	24	F	R	15 years	Right lateral temporal lobe
**S25**	Beijing	Depth	1000	17	F	R	2 years	Periventricular heterotopia
**S26**	Beijing	Depth	2000	19	F	R	8 years	Left medial temporal lobe
**S27**	Beijing	Depth	2000	34	M	R	11 years	Right medial temporal lobe
**S28**	Beijing	Depth	2000	29	F	R	9 years	Right middle frontal
**S29**	Beijing	Depth	2000	20	F	R	3 years	Right medial temporal lobe

**S30**	Beijing	Depth	2000	35	M	R	3 years	Right medial temporal lobe
**S31**	Beijing	Depth	2000	23	F	R	10 years	Right posterior insula/postcentral gyrus

Supplementary Table 1. Subject demographics and characteristics

Subject	# Task Runs	Total Task Duration (mins)	# Rest Runs	Total Rest Duration (mins)	# Total Electrode Sites	# DAN Activated Sites	# SN Activated Sites	# DMN Deactivated Sites
S1	6	32	1	8.95	128	10	2	9
S2	5	30	0	0	98	1	8	1
S3	8	48	2	11.12	136	3	7	5
S4	5	30	0	0	132	0	14	0
S5	4	24	1	7.73	210	5	1	10
S6	8	48	2	11.25	116	4	8	1
S7	5	30	2	11.35	90	2	6	2
S8	4	24	2	17.08	60	6	2	1
S9	4	24	0	0	114	0	8	0
S10	4	24	2	11.81	110	0	6	1
S11	4	24	1	6.5	98	0	5	1
S12	4	32	1	6.5	84	2	5	4
S13	4	32	1	6.5	156	3	5	7
S14	4	32	0	0	100	0	0	0
S15	4	32	1	6.5	96	0	3	2
S16	4	10	1	6.5	146	6	4	6
S17	5	35	0	0	96	1	0	0
S18	5	34	0	0	130	1	8	1
S19	7	43	0	0	88	4	1	2
S20	7	42	1	6.5	142	0	10	15
S21	6	40	1	6.5	172	11	3	4
S22	6	42	0	0	96	7	1	0
S23	5	30	0	0	152	0	0	0
S24	6	36	2	13	94	0	14	4
S25	5	30	2	18.82	182	10	23	1
S26	6	36	0	0	120	12	2	0
S27	5	30	2	13	94	1	1	1
S28	6	38	0	0	48	2	1	0
S29	6	36	2	20.3	120	0	12	8
S30	4	24	2	21.05	152	2	9	6
S31	6	36	0	0	144	0	3	0

Supplementary Table 2. Summary of sessions conducted and task-responsive electrodes identified within networks of interest (“Activated” and “Deactivated” indicate significant HFB increase and decrease, respectively, during target trials in the GradCPT).

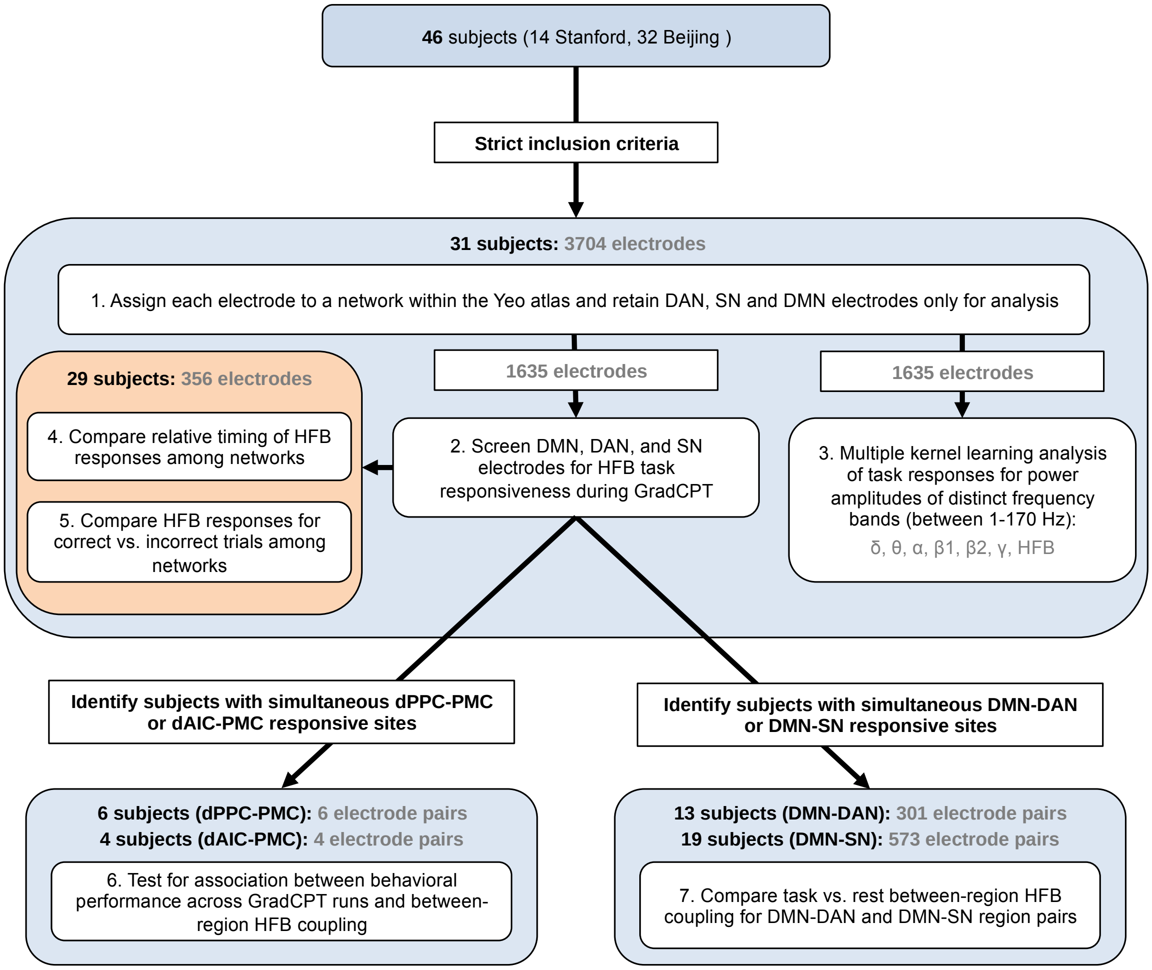

Supplementary Figure 1. Analysis workflow, including the total numbers of subjects and electrodes included for each analysis step.

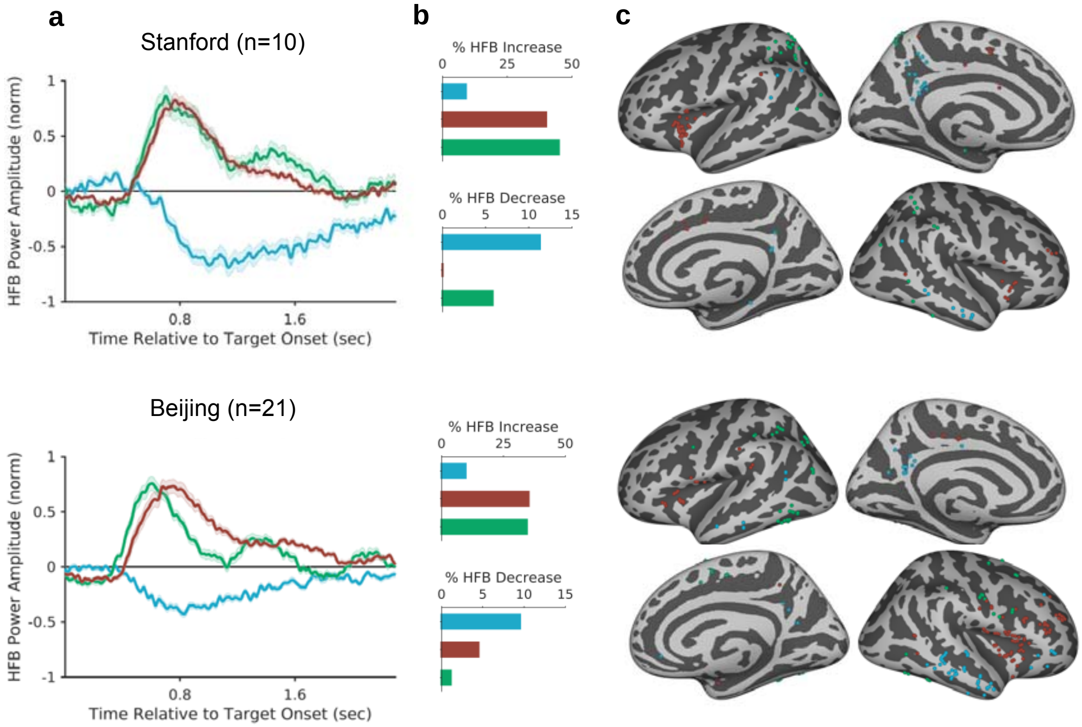

Supplementary Figure 2. Functional localization of electrode sites in the DAN, SN and DMN for Stanford (upper panel) and Beijing (lower panel) cohorts. **a)** High-frequency broadband (HFB; 70-170 Hz) responses during the presentation of mountain (target) stimuli in the GradCPT. Time courses show the grand average of the trial-wise means within all task-responsive electrodes ($p<0.05$, cluster-based permutation test, corrected for multiple comparisons within networks within subjects). **b)** Relative proportions of electrodes within each network showing significant HFB increase (top) and decrease (bottom). **c)** Locations of responsive electrodes within each network (fsaverage space).

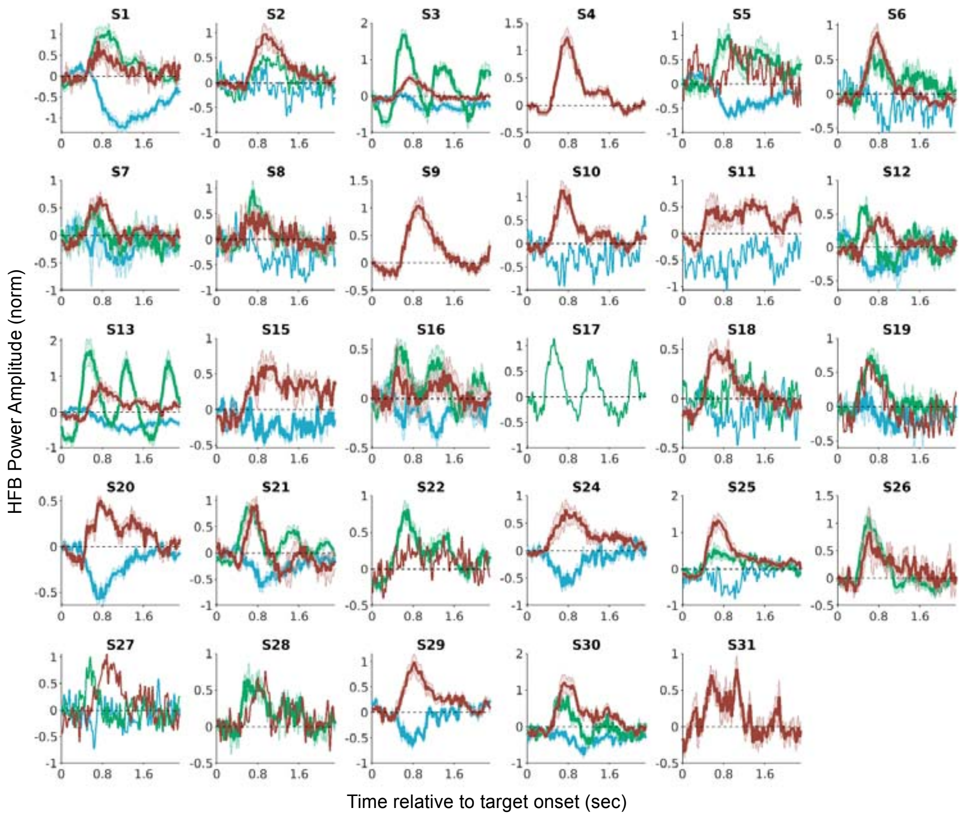

Supplementary Figure 3. Within-subject grand-average HFB responses during the presentation of mountain (target) stimuli in the GradCPT. The means of electrodes with significant HFB increases in the DAN (green), HFB increases in the SN, and HFB decreases in the DMN (blue) are plotted relative to target onset time ($p<0.05$, cluster-based permutation test, corrected for multiple comparisons within networks within subjects). Error bars indicating standard error of the mean are shown only when more than one significant electrode was found within a given network. Plots are shown only for subjects that had at least one electrode with a significant response within at least one of the three networks of interest (29 out of 31 total subjects).

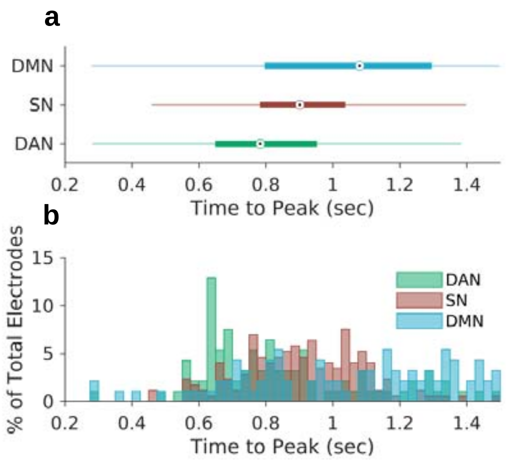

Supplementary Figure 4. Time-to-peak (TTP) of task-evoked HFB responses during commission error trials. a) Box plots showing TTP for all task-responsive electrodes within each network [based on HFB increase (DAN and SN) or HFB decrease (DMN)]. The central point indicates median, box bounds indicate 25th and 75th percentiles, and whiskers indicate most extreme data points not considered to be outliers. **b)** Histogram of the distribution of TTP values for all electrodes. There was a significant interaction between TTP and electrode network assignment ($F_{1,357}$=8.0, p=0.005, F-test on linear mixed effects model). Direct comparisons between network pairs revealed that DAN was earlier than SN ($F_{1,250}$=2.71, p=0.10, F-test on linear mixed effects model), DAN was significantly earlier than DMN ($F_{1,184}$=33.9, p=2.4x10^{-8}), and SN was significantly earlier than DMN ($F_{1,264}$=9.41, p=0.002, F-test on linear mixed effects model).
