# Supplementary material for: Electrophysiological dynamics of antagonistic brain networks reflect attentional fluctuations
Source: Nat Commun. 2020 Jan 16;11:325. doi: 10.1038/s41467-019-14166-2 (PMC6965628; doi:10.1038/s41467-019-14166-2)
Supplement: Supplementary file 2 — Reporting Summary [file 41467_2019_14166_MOESM2_ESM.pdf]

nature research

Corresponding author(s): Josef Parvizi
Last updated by author(s): Nov 6, 2019

Reporting Summary

Nature Research wishes to improve the reproducibility of the work that we publish. This form provides structure for consistency and transparency in reporting. For further information on Nature Research policies, see Authors & Referees and the Editorial Policy Checklist .

Statistics

For all statistical analyses, confirm that the following items are present in the figure legend, table legend, main text, or Methods section.

n/a	Confirmed	
☐	☒	The exact sample size (n) for each experimental group/condition, given as a discrete number and unit of measurement
☐	☒	A statement on whether measurements were taken from distinct samples or whether the same sample was measured repeatedly
☐	☒	The statistical test(s) used AND whether they are one- or two-sided<br>*Only common tests should be described solely by name; describe more complex techniques in the Methods section.*
☐	☒	A description of all covariates tested
☐	☒	A description of any assumptions or corrections, such as tests of normality and adjustment for multiple comparisons
☐	☒	A full description of the statistical parameters including central tendency (e.g. means) or other basic estimates (e.g. regression coefficient) AND variation (e.g. standard deviation) or associated estimates of uncertainty (e.g. confidence intervals)
☐	☒	For null hypothesis testing, the test statistic (e.g. F, t, r) with confidence intervals, effect sizes, degrees of freedom and P value noted<br>*Give P values as exact values whenever suitable.*
☒	☐	For Bayesian analysis, information on the choice of priors and Markov chain Monte Carlo settings
☒	☐	For hierarchical and complex designs, identification of the appropriate level for tests and full reporting of outcomes
☒	☐	Estimates of effect sizes (e.g. Cohen's d, Pearson's r), indicating how they were calculated

Our web collection on statistics for biologists contains articles on many of the points above.

Software and code

Policy information about availability of computer code

Data collection	Custom Matlab code was used for presentation of visual stimuli during data collection
Data analysis	Custom Matlab code was used for analyses of MRI and intracranial EEG data. The custom code drew from tools in Freesurfer v6.0, iElvis, SPM12, and Fieldtrip 20160615.

For manuscripts utilizing custom algorithms or software that are central to the research but not yet described in published literature, software must be made available to editors/reviewers. We strongly encourage code deposition in a community repository (e.g. GitHub). See the Nature Research guidelines for submitting code & software for further information.

Data

Policy information about availability of data

All manuscripts must include a data availability statement. This statement should provide the following information, where applicable:

- Accession codes, unique identifiers, or web links for publicly available datasets
- A list of figures that have associated raw data
- A description of any restrictions on data availability

The data that support the findings of this study are available from the corresponding author on reasonable request.

Field-specific reporting

Please select the one below that is the best fit for your research. If you are not sure, read the appropriate sections before making your selection.

☒ Life sciences ☐ Behavioural & social sciences ☐ Ecological, evolutionary & environmental sciences

Life sciences study design

All studies must disclose on these points even when the disclosure is negative.

Sample size	Sample size was chosen based on pilot data and previous studies demonstrating that single-subject analyses yield significant task-evoked responses within the brain regions of interest.
Data exclusions	Subjects were excluded based on pre-established exclusion criteria : 1) poor behavioral performance (as described in the manuscript) that could indicate lack of compliance with task instructions or inability to perform the task successfully (n=12); 2) major structural brain abnormalities that impeded MRI-based cortical surface reconstruction, including encephalomalacia and damage from prior resections (n=3).
Replication	The primary results were replicated in Stanford versus Beijing cohorts (see Supplementary Figure 2).
Randomization	N/A
Blinding	N/A

Reporting for specific materials, systems and methods

We require information from authors about some types of materials, experimental systems and methods used in many studies. Here, indicate whether each material, system or method listed is relevant to your study. If you are not sure if a list item applies to your research, read the appropriate section before selecting a response.

Materials & experimental systems

n/a	Involved in the study	
☒	☐	Antibodies
☒	☐	Eukaryotic cell lines
☒	☐	Palaeontology
☒	☐	Animals and other organisms
☐	☒	Human research participants
☒	☐	Clinical data

Methods

n/a	Involved in the study	
☒	☐	ChIP-seq
☒	☐	Flow cytometry
☒	☐	MRI-based neuroimaging

Human research participants

Policy information about studies involving human research participants

Population characteristics	The study was conducted in male and female patients with refractory focal epilepsy.
Recruitment	Patients undergoing intracranial electrode monitoring, as part of clinical procedures, volunteered to participate in the research.
Ethics oversight	Stanford Institutional Review Board and Medical Ethics Committee of Beijing Tian Tan Hospital

Note that full information on the approval of the study protocol must also be provided in the manuscript.
